# Efficient labelling for efficient deep learning: the benefit of a multiple-image-ranking method to generate high volume training data applied to ventricular slice level classification in cardiac MRI

**DOI:** 10.21037/jmai-22-55

**Published:** 2023-04

**Authors:** Sameer Zaman, Kavitha Vimalesvaran, James P. Howard, Digby Chappell, Marta Varela, Nicholas S. Peters, Darrel P. Francis, Anil A. Bharath, Nick W. F. Linton, Graham D. Cole

**Affiliations:** 1 National Heart and Lung Institute, Imperial College London, London, UK; 2 Imperial College Healthcare NHS Trust, London, UK; 3 AI for Healthcare Centre for Doctoral Training, Imperial College London, London, UK; 4 Department of Bioengineering, Imperial College London, London, UK

**Keywords:** Deep learning (DL), convolution neural network (CNN), data labelling, ground truth, clinical imaging

## Abstract

**Background:**

Getting the most value from expert clinicians’ limited labelling time is a major challenge for artificial intelligence (AI) development in clinical imaging. We present a novel method for ground-truth labelling of cardiac magnetic resonance imaging (CMR) image data by leveraging multiple clinician experts ranking multiple images on a single ordinal axis, rather than manual labelling of one image at a time. We apply this strategy to train a deep learning (DL) model to classify the anatomical position of CMR images. This allows the automated removal of slices that do not contain the left ventricular (LV) myocardium.

**Methods:**

Anonymised LV short-axis slices from 300 random scans (3,552 individual images) were extracted. Each image’s anatomical position relative to the LV was labelled using two different strategies performed for 5 hours each: (I) ‘one-image-at-a-time’: each image labelled according to its position: ‘too basal’, ‘LV’, or ‘too apical’ individually by one of three experts; and (II) ‘multiple-image-ranking’: three independent experts ordered slices according to their relative position from ‘most-basal’ to ‘most apical’ in batches of eight until each image had been viewed at least 3 times. Two convolutional neural networks were trained for a three-way classification task (each model using data from one labelling strategy). The models’ performance was evaluated by accuracy, F1-score, and area under the receiver operating characteristics curve (ROC AUC).

**Results:**

After excluding images with artefact, 3,323 images were labelled by both strategies. The model trained using labels from the ‘multiple-image-ranking strategy’ performed better than the model using the ‘one-image-at-a-time’ labelling strategy (accuracy 86% *vs.* 72%, P=0.02; F1-score 0.86 *vs.* 0.75; ROC AUC 0.95 *vs.* 0.86). For expert clinicians performing this task manually the intra-observer variability was low (Cohen’s κ=0.90), but the inter-observer variability was higher (Cohen’s κ=0.77).

**Conclusions:**

We present proof of concept that, given the same clinician labelling effort, comparing multiple images side-by-side using a ‘multiple-image-ranking’ strategy achieves ground truth labels for DL more accurately than by classifying images individually. We demonstrate a potential clinical application: the automatic removal of unrequired CMR images. This leads to increased efficiency by focussing human and machine attention on images which are needed to answer clinical questions.

## Introduction

A typical cardiac magnetic resonance (CMR) imaging scan for a single patient may contain over a thousand images (1). This number of individual images presents a challenge, even for experienced clinicians, to manually sift through and find the images most relevant to the clinical question being considered. Deep learning (DL) can be used to streamline clinical workflow by automatically detecting features from CMR images to focus clinical attention and determine scanning workflow in real-time (2). However, a major rate-limiting step in the development pipeline for training networks on specific tasks is the significant expert investment of human time required to generate sufficient high-quality labelling on a large enough scale for training (3). Since more data labelling is usually not possible due to expert unavailability, researchers instead focus their efforts on tweaking the model’s architecture and parameters to boost algorithmic performance. Maximising the data yield from limited expert labelling time is rarely prioritised and there is no precedent for the optimal data labelling strategy for clinical images. In medical imaging, the number of clinicians with sufficient experience is limited, meaning that inefficient labelling also has an opportunity cost of stopping clinicians from other useful activities, such as direct clinical care (4). Efficient DL requires an efficient labelling strategy, so it is of utmost importance to ensure labelling humans’ limited time results in the best possible algorithmic performance.

When expert clinicians are labelling medical images, one way to present the information is as single images about which the clinician is asked a binary question. Another is to present a large number of images in parallel and ask clinicians to rank a single feature (which may provide the answer to the binary question) from the group simultaneously presented. The optimally efficient strategy for a team of expert clinicians to label large volumes of medical image data is not known.

We aim to develop a strategy for medical image annotation that results in more secure labels, which take into account the varied opinion of multiple experts rather than the single opinion of one expert. We aim for this strategy to produce labels that result in better performing DL models when the amount of data labelled and the time spent on labelling is the same as other strategies.

**Figure 1:**
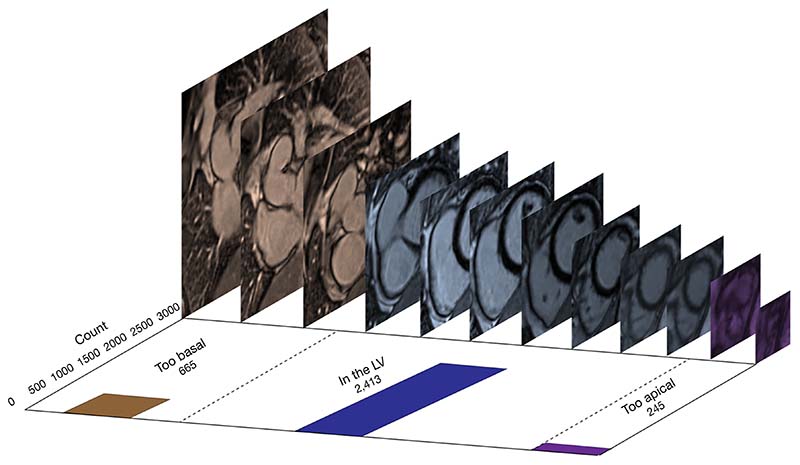
Slices from a stack of short-axis late gadolinium images for a patient in our dataset. Distribution of ‘too basal’, ‘in the LV’, and ‘too apical’ classes in the dataset of 3,323 images. Classifications labelled using the ‘one-image-at-a-time’ strategy. LV, left ventricle.

In this study we compare two ways of labelling training data for machine learning (ML) in clinical imaging: (I) a strategy of single assessments into one of three discrete categories and (II) a ranking strategy of simultaneous comparisons of multiple images. We hypothesise that, given the same amount of expert labelling time, a DL classifier trained using data from a ‘multiple-image-ranking’ labelling strategy outperforms the same model trained with data from a single image assessment labelling strategy. We apply this in CMR to the paradigm of identifying the subset of late gadolinium short-axis cardiac views that contain the heart by comparing the algorithmic performance of DL classifiers trained using both labelling strategies. This is clinically important because these images are used to examine the heart for scar [late gadolinium enhancement (LGE)] due to a range of conditions (5–7). The identification and characterization of LGE, especially in the left ventricle (LV) is a key determinant of treatment (8). When a stack of short-axis LGE images is acquired only some of the slices image the LV, which is relevant to clinicians assessing LGE. Many images are ‘too basal’ or ‘too apical’; these are not relevant for LGE assessment and may divert clinicians’ focus from the clinical question (Figure 1). Using ML to automate slice level classification in order to streamline LGE assessment workflow is an area of active research (9,10). In a future with both clinician and artificial intelligence (AI) image interpretation, algorithms such as the classifier presented in this study could automatically remove images in which the structure of interest is not present, focusing human and machine attention to only review the images that maximize clinical yield. We present the following article in accordance with the STARD reporting checklist (available at https://jmai.amegroups.com/article/view/10.21037/jmai-22-55/rc).

## Methods

### Data extraction and pre-processing

Anonymised short-axis late gadolinium images for 300 scans (3,552 individual images) performed at our centre in London, UK between 2018 and 2022 were retrospectively and randomly extracted from our local CMR database [dataset (11)]. The eligibility criteria were having had a CMR with short-axis late gadolinium imaging between 2018 and 2022. The study was conducted in accordance with the Declaration of Helsinki (as revised in 2013). The study was approved by the ethics board of the UK Health Regulatory Agency (Integrated Research Application System identifier 243023) and informed consent was waived due to the data and analyses being anonymised at source. Two experienced CMR clinicians reviewed the extracted images and excluded those of insufficient quality due to significant artefact. Each short-axis stack was separated into its constituent individual images, which were randomly shuffled prior to manual labelling. The images were shuffled to ensure that labellers were ranking different slices from different scans rather than simply reordering all the slices from a single scan. This enabled the identification of generic inflection points between apical, LV and basal slices across the entire dataset.

### Data labelling and curation

A consensus meeting of clinicians determined definitions for the labelling of LV slice level according to the Society of Cardiovascular Magnetic Resonance (SCMR) guidelines (12):

(I) ‘Too basal’: a slice in which there is LV myocardium visible around less than 50% of the circumference of the blood pool;
(II) ‘LV’: a slice in which there is LV myocardium visible around more than 50% of the circumference of the blood pool;
(III) ‘Too apical’: a slice beyond the LV apex in which no LV myocardium is visible.

**Figure 2:**
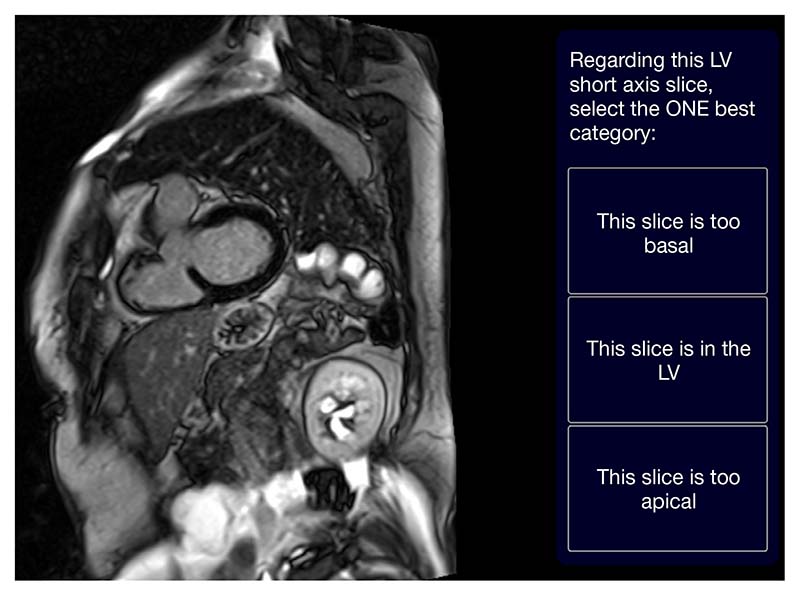
A screenshot of the ‘one-image-at-a-time’ data labelling strategy using the Unity platform. LV, left ventricle.

The images were uploaded to the Unity Imaging platform for labelling (13). Cardiac imaging experts performed the labelling (SZ, KV, GDC). Two strategies of ground truth labelling were performed, in parallel, on the same dataset of images:

Strategy 1: ‘one-image-at-a-time’. Single images were presented to the labeller sequentially. The labeller was instructed to review the image and select only one of the following options: “this slice is too basal”, “this slice is in the LV”, or “this slice is too apical” (Figure 2). There were three clinicians labelling the pool of images but a single image was only labelled by a single labeller and then removed from the pool (i.e., not repeated).

**Figure 3:**
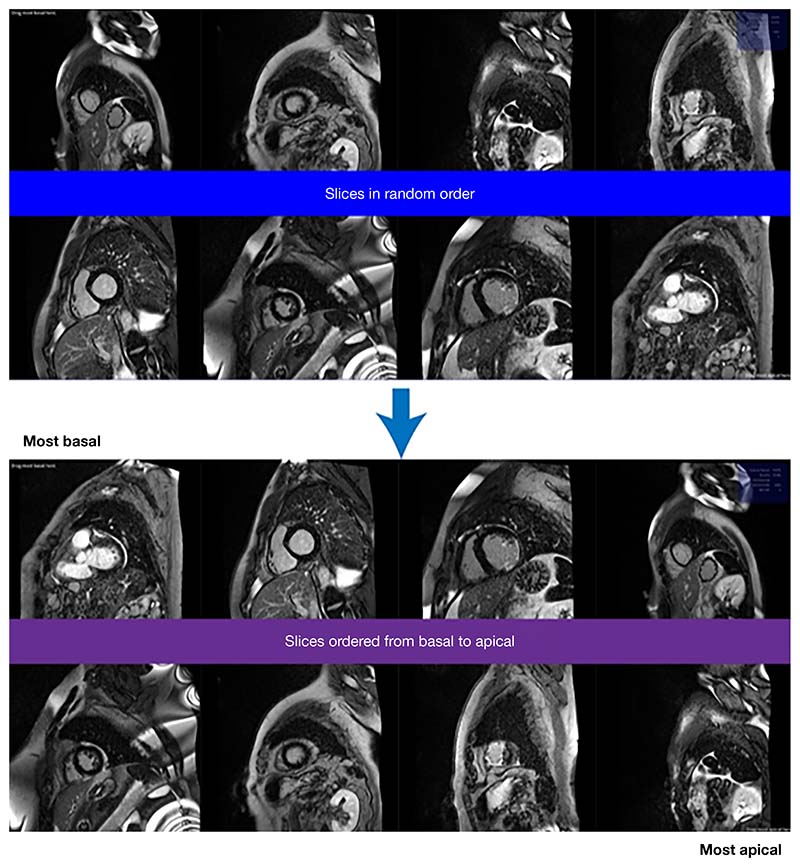
A screenshot of ‘multiple-image-ranking’ data labelling strategy, before and after images were ranked using the Unity imaging platform.

Strategy 2: ‘multiple-image-ranking’. Shuffled images were presented to labellers in batches of eight in random order. Labellers were instructed to reorder the images by moving the image tiles on the screen (e.g., click and drag) from “most basal” to “most apical” (Figure 3).

A bespoke algorithm was designed to determine which images were presented for labelling. This algorithm measured four parameters:

(I) ‘Ranking’: the ordinal position of the image within the entire dataset from 1 (most basal image) to 3,323 (most apical image). This was initialized randomly at the start;
(II) ‘Rating’: a score between 0 (very apical) and 3,000 (very basal) that was initialized at 1,500 for all images;
(III) ‘View count’: the number of times a particular image had been viewed and labelled;
(IV) ‘Volatility’: a measure of the agreement between multiple ratings of the same image, taking values between 0 (labellers always disagree about the position of this image in the dataset) and 1 (labellers agree perfectly about the position of this image in the dataset). Initialised at 0 for all images.

These variables were defined and updated in the back-end of the ranking algorithm, and were never shown to the raters. The only thing the raters saw were batches of 8 images which they could drag from most basal (top left of screen) to most apical (bottom right of screen), and a button to submit the current batch and load the next batch (Figure 3).

At the start, eight random images were presented. After the labeller ranked and submitted them, the ‘rating’, ‘view count’, and ‘volatility’ was updated for those eight images, and the ‘ranking’ was updated for the entire dataset. Images that were labelled as being towards the basal end of the spectrum had their ‘rating’ increase (closer to 3,000), those labelled as being towards the apical end of the spectrum had their ‘rating’ decrease (close to 0), whilst those regarded as being in the LV had the ‘rating’ stay close to 1,500 (the starting value). If an image had been previously rated as being quite basal, but a new labeller rated it as quite apical (i.e., a very divergent opinion) there would be larger jump in the rating and the volatility would increase (and vice versa if the new labeller agreed with the previous labeller’s label).

Then, a new set of eight images was presented to the labeller. Each time a labeller submitted an ordered batch, the four parameters of the algorithm were updated in real-time. The algorithm prioritised images for review on the following criteria:

(I) Images that had not been seen before (view count =0), or had been reviewed much fewer times than other images in the volume were preferentially shown;
(II) Images with high volatility (seen multiple times but different opinions between labellers) were preferentially shown until the volatility of their label decreased.

Images continued to be shown until every image had been reviewed at least three times. Three expert labellers worked independently on the pooled dataset but because of representation, were sometimes viewing images that had been seen by other labellers, contributing to the dynamic ratings in real-time. The resulting final dataset was an ordinal ranking of images based on slice level (rank 1 = most basal; rank 3,323 = most apical), based on the pooled labelling effort of three experts.

To enable direct comparison between the two versions of the model (trained using labels from the ‘one-image-at-a-time’ and ‘multiple-image-ranking’ strategies), discrete labels had to be assigned to the ‘multiple-image-ranking’ labels to reflect the same three classes labelled using the ‘one-image-at-a-time’ strategy. After the ranking had been completed, the images were probed to determine the inflection points at which ‘too basal’ transitioned to ‘LV’ and at which ‘LV’ transitioned to ‘too apical’. The discrete labels of ‘too basal’ and ‘too apical’ were assigned above and below these thresholds respectively, with ‘in the LV’ being assigned to all the images in between.

To enable parity of labelling effort, the cumulative labelling time for each method was standardised at 5 hours. The labelling was performed by SZ, KV, and GDC. They were blinded to each other’s labelling.

### Neural network design and training

The labelled data were split into training, validation, and test sets (80:10:10) using a stratified method to reflect class imbalances. The architecture chosen was an adapted version of Resnet (14) (trained from scratch starting with random weights) with a feed-forward network and final layer that output the probability of the input image being “too basal”, “in the LV”, or “too apical”. The class with the highest output probability was assigned as the predicted label for that image.

Two versions of the same network were trained; one for each labelling strategy (one-image-at-a-time or multiple-image-ranking). For the training and validation sets, the labels assigned by the respective labelling strategy were retained. For the test set, only the labels assigned by ‘one-image-at-a-time’ strategy were used as the definitive ground truth for performance evaluation. The test set was not seen by the network during training and validation.

Training was augmented by random crops to a 224×224 pixel size, flips and rotations. For the training set only, images were first cropped 40% top/bottom and 30% left/ right in order to remove non-cardiac structures from the image periphery. This resulted in rectangular images which were made square by applying a 256×256 pixel centre crop. After this crop the random crop to 224×224 pixels was applied for augmentation. Each version underwent 10 training runs starting with different random weight initialization. The models were trained on a Tesla P100-PCIE-16GB GPU. Model parameters are shown in Table 1.

### Evaluation of performance

Performance of both versions was evaluated on the same test set, using labels assigned by the one-image-at-a-time strategy. We regarded these labels to be the definitive ground truth because the one-image-at-a-time strategy is the currently established labelling paradigm for these type of data. The following performance metrics were calculated for both versions: accuracy, F1-score and area under the receiver operating characteristics curve (ROC AUC).

### Statistical analysis

For statistical comparison between the two versions, the ‘too basal’ and ‘too apical’ predictions were combined into a single class called ‘not in the LV’. The predictions could therefore be evaluated as a binary classification (‘in the LV’ *vs.* ‘not in the LV’) enabling comparison with McNemar’s tests (15). The null hypothesis was that there was no difference in the overall accuracy between the model trained using the ‘one-image-at-a-time’ strategy and the model trained using the ‘multiple-image-ranking’ strategy. A P value <0.05 was considered significant.

### Intra-rater and inter-rater variability

The test set (333 images, 10% of the dataset) was double-labeled by the same clinician and labeled by a second experienced clinician at least 2 weeks apart, in a blinded manner, using the one-image-at-a-time labelling strategy. Cohen’s κ was calculated to assess intra- and inter-rater variability for each labelling class.

## Results

A total of 229 images were removed during quality control due to artefact, leaving 3,323 short-axis late gadolinium images available for labelling. Experienced clinicians were given a maximum of 5 hours (cumulative) per method to label as many images as possible using the two labelling strategies:

υ ‘One-image-at-a-time’: after 5 hours clinicians had assigned labels to the entire dataset of 3,323 images. Although there were three labellers working on the dataset, each image was only labeled once by a single labeller and was not shown again.
υ ‘Multiple-image-ranking’: three clinicians labelled for a combined total 5 hours (SZ—2 hours; KV—2 hours; GDC—1 hour). Every image in the dataset of 3,323 had been viewed and ranked at least 3 times. A total of 44,097 individual pairwise comparisons were made.

The distribution of classes from the ‘one-image-at-a-time’ strategy is shown in Figure 1.

### Label agreement between the two labelling strategies

**Figure 4:**
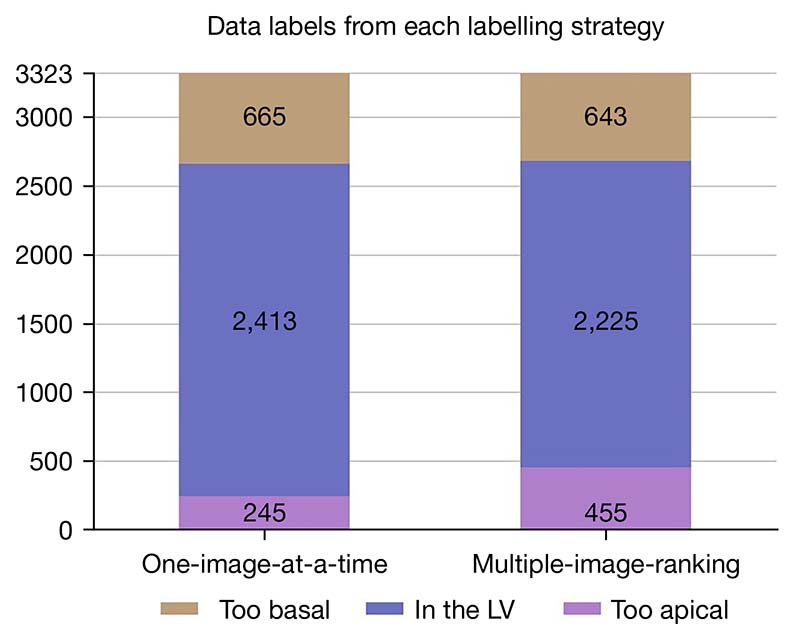
Data labels from each labelling strategy. LV, left ventricle.

The data labels arising from each labelling strategy are illustrated in Figure 4. The multiple-image-ranking strategy labelled fewer images in the ‘LV’ class (2,225 *vs.* 2,413) and more in the ‘too apical’ class (455 *vs.* 245) than the one-image-at-a-time strategy. The number of images labelled in the ‘too basal’ class were comparable between the two strategies (one-image-at-a-time =665; multiple-image-ranking =643). Overall, there was only moderate agreement between the labels from the two strategies (Cohen’s κ=0.67).

### Model performance

**Figure 5:**
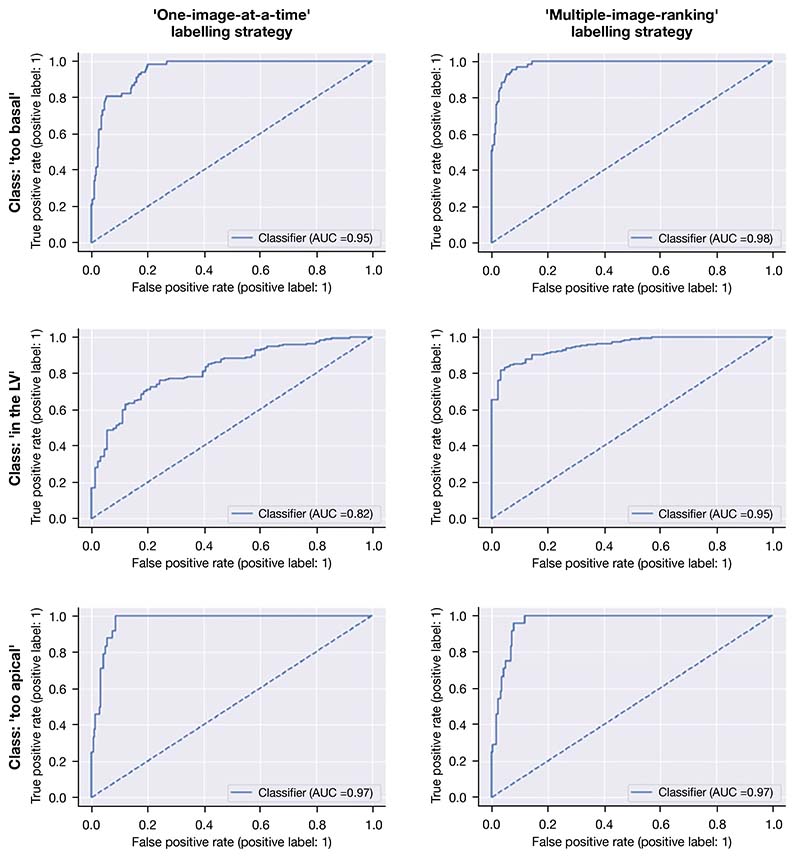
ROCs curves of two versions of the model evaluated on the test set of 333 images. AUC, area under the curve; LV, left ventricle; ROC, receiver operating characteristic.

Both versions of the model (with labels from the ‘one-image-at-a-time’ or ‘multiple-image-ranking’ labelling strategies) were trained with the same parameters for 70 epochs. For each version, the iteration with the smallest loss on the validation set was selected. Accuracy, F1-score, and ROC AUC on the test set are shown in Table 2. The ROC curves and AUC for both model versions are shown in Figure 5.

### Comparison of model versions

Confusion matrices of each model’s agreement with the ground-truth test set of 333 images is shown in Table 3. McNemar’s test showed that the ‘multiple-image-ranking’ model’s predictions had significantly higher agreement with the test set labels than the ‘one-image-at-a-time’ model (P=0.02) (Table 4).

### Intra-rater and inter-rater variability

On the test set of 333 images that were double-reported by the same expert and another expert, the intra-observer variability was low (Cohen’s κ=0.90), but the inter-observer variability was higher (Cohen’s κ=0.77) (Table 5).

## Discussion

This study has two key findings. First, we demonstrate that a ‘multiple-image-ranking’ strategy is feasible for ground truth labelling of clinical image data. For a fixed human time-investment, a network trained from this strategy was superior to a network trained with a ‘one-image-at-a-time’ labelling strategy (P=0.02). Second, we present a clinical application of this strategy to automatically detect which CMR short-axis slices are not imaging the LV myocardium with high accuracy (86%). This enables automatic removal of unrequired images to focus clinicians’ attention on the images most relevant to answering clinical questions.

### Comparison with other approaches

Since annotated medical data are a limited resource due to limited availability of expert clinical labellers, trying to maximise algorithmic performance whilst reducing labelling effort is an area of active research in ML. One approach is transfer learning using networks that have been pre-trained on huge datasets in an unsupervised fashion and then fine-tuning for the specific task using a few (much fewer than if training from scratch) manually annotated examples (16). Another approach is ‘active learning’, in which the examples within the dataset that are most likely to contribute to network learning are selected for manual annotation (17,18). This can be combined with metrics of label uncertainty to identify the data that will yield the most performance-value from manual annotation (19). A third approach is to use contrastive learning, a variant of self-supervised learning, based on the intuition that transformations of an image should have similar representations to each other and the original image, but dissimilar representations to different images. These approaches have shown some success to get the most algorithmic training value from limited clinical images (20,21). All three approaches aim to reduce the amount of data that needs to be manually labelled, but they do not enable the contribution of multiple different labellers with minimal time penalty. Our multiple-image-ranking approach enables the entire data volume to be viewed multiple times, by multiple experts, without taking more time than the one-image-at-a-time strategy. This approach reflects the wide spectrum of individual labelling behaviour and reduces label volatility without having to label fewer data or spend more time labelling. Our approach may be particularly helpful for tasks in which there are no clear class boundaries and there is intrinsic intra- and inter-rater variability.

### Impact on clinical ground-truth labelling

Researchers in clinical AI assiduously hone algorithmic performance by tweaking model architecture, but optimising the data labelling strategy is rarely prioritised. There is no precedent for the optimal data labelling strategy for clinical images. In this study we present a novel strategy to streamline the process of creating ground truth labels by clinician experts for ML, and compare it to a conventional labelling approach. The version of our classifier trained using the ‘multiple-image-ranking’ strategy significantly outperformed the version trained using the ‘one-image-at-a-time’ strategy. Since the same number of images were labelled and the same time spent on both strategies, the differences between the two models’ performance is not due to data amount or labelling effort. The ‘multiple-image-ranking’ strategy may have succeeded for a number of reasons. First, it shows many pictures in batches so a single ranking enables multiple pairwise comparisons with relatively little time penalty. Ranking a batch of eight images results in 28 unique pairs of comparisons, and it is efficient because it does not necessarily require systematic comparison of every pair. For example, image A and C do not need to be compared explicitly if both A and B, and B and C have already been compared. Ranking also allows reinspection of the same images, and correction of spurious errors which may have occurred on the first assessment. A conventional ‘one-image-at-a-time’ approach does not allow this unless all images are re-reviewed.

Although our analyses show that experts are usually internally consistent in this task (Cohen’s κ=0.90), there is more disagreement between experts (Cohen’s κ=0.77). AI algorithms trained on labels from a single operator may be less secure than those which are developed from a broader spectrum of expert opinion. The key differences between the methods are summarized in Table 6.

For the same human labelling time investment, the version trained on the labels from the ‘multiple-image-ranking’ strategy outperformed the ‘one-image-at-a-time’ strategy, demonstrating it is a more efficient way to harness human time. This is quite a surprising finding since the test set was labelled using the one-image-at-a-time strategy. We hypothesise this is because the multiple-image-ranking strategy reframes the labelling task from classification to regression. This has at least two potential advantages. First, a slice which is borderline between “too basal” and “in the LV” is no longer forced into a dichotomous label, with the model being punished for getting it wrong. Instead, the model is rewarded for placing the slice appropriately on the decision boundary. The influence of ‘grey zone’ labels and the problems it causes for classification tasks is a well recognised phenomenon. Indeed, our multiple-image-ranking strategy could be regarded to be a novel extension of categorical label smoothing (22). In this type of strategy, the classification boundaries are blurred and performance improvements are significant. This may be particularly useful for datasets where there is controversy at the boundaries between classes, such as LV slice level as illustrated by the inter-observer variability.

Second, our multiple-image-ranking strategy has in built robustness to labelling errors, whilst allowing collaborative labelling with pooling of experience. The multiple-image-ranking strategy relies on rapid repeated relative labelling, therefore rare stochastic labelling errors, or unrepresentative expert opinions, have only a modest influence over the final rating for a given image. We propose these two phenomena may be responsible for the improved performance of using the multiple-image-ranking strategy for model training. Further work is required to test and prove these hypotheses formally.

We recommend this method for ordinal data that does not require feature segmentation. Within CMR, this method could be used for other tasks such as quantifying the amount of LGE, for which current methods are imprecise and time-consuming (23), hence preventing automation by ML. Streamlining data labelling by clinical experts addresses a major bottleneck in the clinical ML pipeline (24).

### Impact on clinical workflow

Using AI algorithms to streamline radiographer workflow and guide sequence selection in CMR has been previously described (2). This study is another proof of concept of AI technology to improve clinical workflow in imaging. CMR studies frequently exceed 1,000 individual images; datasets of this magnitude are challenging even for experienced clinicians to manually sift through. Automatic removal of images that are irrelevant to a clinician’s analysis of the study (such as the removal by our classifier of short-axis slices that do not image the LV) would help clinicians to focus their attention on images relevant to answering the clinical question.

In our dataset of 3,324 images, 664 (20.0%) were too basal and 246 (7.4%) too apical. These short-axis LGE images did not image the LV and so were unhelpful to clinicians undertaking scar analysis. Automatically removing these images from the dataset reduced the size of CMR studies by over 25%.

Automatic image curation could be used to focus not only human attention, but also the machine attention of other AI algorithms in the imaging workflow. For example in CMR, algorithms that aid humans by automatically segmenting cardiac structures to derive volumetric and functional measurements still require manual clinician input to edit the segmentation when the slice is not imaging the LV. The need for this manual editing could be mitigated by automatically removing the non-LV slices before the segmentation algorithm, using a classifier like the one we present in this study. Another challenge for automation in CMR is the quantification of LGE, for which semi-automated methods are available but not routinely used in clinical practice because they require time-consuming human-computer interaction including determination of which slices are imaging the LV (9,23). Automatic removal of the non-LV slices would streamline LGE quantification and standardize reporting of this important imaging biomarker.

### Limitations

Our study has some limitations. First, neither version of the model is 100% accurate for all three classes. Like many classifications in clinical medicine, determining the slice level in CMR requires some subjective judgement and in our study, clinicians did not always agree with each other. Our inter-rater experiment illustrates that there is intrinsic variability between clinicians for this task (inter-rater Cohen κ=0.77). The version trained using the ‘multiple-image-ranking’ strategy agreed with the clinicians’ labels more than clinicians agreed with themselves, but this was not the case for the ‘one-image-at-a-time’ labelling strategy; the innovation of ‘multiple-image-ranking’ lifted the algorithm to supra-human performance. This further illustrates the benefit of using labels generated by a group of experts to improve label (and algorithm) accuracy.

Second, this study is proof of concept of this labelling strategy. For the ‘LV’ class in particular, there was a trade-off between recall and precision. This was exacerbated by significant class imbalances in the data. We favored a higher recall because the clinical impact of a false negative (keeping a non-LV slice in the dataset) is more acceptable than of a false positive (wrongly removing an LV slice from the dataset).

Third, as with all DL models, it is possible that our findings may not generalize to other settings due to ‘overfitting’ (25). To mitigate this, we report performance on a test set that was only used after training the models. Furthermore, the dataset was assembled from randomly selected scans performed at two different hospitals across a number of years. Our future work will test the reproducibility of these results by evaluating the models’ performance on large, public CMR datasets.

## Conclusions

We present a novel strategy for ground truth labelling of medical image training data for ML. Comparing multiple images side-by-side using the ‘multiple-image-ranking’ strategy obtains information from human image analysis more efficiently than by classifying them individually. We present proof of concept that this labelling strategy results in a trained classifier for LV slice level categorisation that outperforms a ‘one-image-at-a-time’ labelling strategy, and with higher accuracy than the intrinsic inter-human variability for this task. A potential clinical application of this is the automatic removal of unrequired CMR images. This leads to increased efficiency by focussing human and machine attention on images which are needed to answer clinical questions.

## Figures and Tables

**Table 1:** Key training parameters of DL classifier

Parameters	Value
Architecture	Resnet + feed forward neural network
Train:validation:test	80:10:10
Trainable parameters	22,199,747
Optimizer	Adam with weight decay
Learning rate	1e-4

DL, deep learning.

**Table 2:** Performance of two versions of the trained model (trained using labels from the ‘one-image-at-a-time’ labelling strategy or the ‘multiple-image-ranking’ labelling strategy) on a test set of 333 images

Performance metric	‘One-image-at-a-time’ labelling strategy	‘Multiple-image-ranking’ labelling strategy
Class: ‘too basal’		
Accuracy (95% CI)	0.91 (0.87–0.94)	0.93 (0.90–0.96)
ROC AUC (95% CI)	0.95 (0.91–0.99)	0.98 (0.96–1.00)
F1-score	0.77	0.85
Class: ‘in the LV’		
Accuracy (95% CI)	0.72 (0.67–0.77)	0.86 (0.82–0.89)
ROC AUC (95% CI)	0.82 (0.77–0.87)	0.95 (0.93–0.97)
F1-score	0.78	0.90
Class: ‘too apical’		
Accuracy (95% CI)	0.80 (0.75–0.84)	0.92 (0.88–0.95)
ROC AUC (95% CI)	0.97 (0.92–1.00)	0.97 (0.92–1.00)
F1-score	0.42	0.63
Overall		
Accuracy (95% CI)	0.72 (0.67–0.77)	0.86 (0.82–0.89)
ROC AUC (95% CI)	0.86 (0.82–0.90)	0.95 (0.93–0.97)
F1-score	0.75	0.86

CI, confidence interval; ROC AUC, area under the receiver operating characteristics curve; LV, left ventricular.

**Table 3:** Confusion matrices for two model versions trained using data from either the ‘one-image-at-a-time’ or ‘multiple-image-ranking’ labelling strategy, to predict whether images in the test set of 333 images are ‘in the LV’ or ‘not in the LV’

Model name	Predictions (n)	Model prediction: in the LV	Model prediction: not in the LV
One-image-at-a-time	Ground truth (test set): in the LV	167	75
	Ground truth (test set): not in the LV	18	73
Multiple-image-ranking	Ground truth (test set): in the LV	202	40
	Ground truth (test set): not in the LV	6	85

LV, left ventricle.

**Table 4:** Recall, precision, and accuracy of two versions of the trained model, tested on a set of 333 images

Predictions from	Recall (95% Cl)	Precision (95% Cl)	Accuracy (95% Cl)	P value (vs. ‘one-image-at-a-time’)
‘One-image-at-a-time’ labelling strategy	0.90 (0.85–0.94)	0.49 (0.41–0.58)	0.72 (0.67–0.77)	–
‘Multiple-image-ranking’ labelling strategy	0.97 (0.94–0.99)	0.68 (0.59–0.76)	0.86 (0.82–0.90)	0.02

P value from McNemar’s test between the ‘one-image-at-a-time’ version and the ‘multiple-image-ranking’ version. Cl, confidence interval.

**Table 5:** Intra-rater and inter-rater variability for LV slice level classification

Slice level/rater variability	Rater 1, pass 1	Rater 1, pass 2	Rater 2
Too basal (n)	66	56	47
In the LV (n)	242	255	257
Too apical (n)	25	22	29
Variability vs. rater 1 pass 1 (Cohen’s κ)	–	0.90	0.77

LV, left ventricle.

**Table 6:** Features of the two ground-truth labelling strategies

Features	One-image-at-a-time labelling strategy	Multiple-image-ranking labelling strategy
Total labelling hours (sum of all experts’ time)	5	5
Number of experts contributing	1	3
Number of views per image	1	>3
Number of images labelled	3,552	3,552
Paired image comparisons	0	44,097
Labelling output	Unrelated classes	Ordinal list

## Highlight box

### Key findings

- A deep learning (DL) classifier for left-ventricular slice position of magnetic resonance (CMR) images was designed.
- The version of the model trained using labels from a novel efficient data labelling strategy called ‘multiple-image-ranking’ had significantly higher overall accuracy than a version trained using the current paradigm of labelling ‘one-image-at-a-time’ (accuracy 86% *vs.* 72%, P=0.02).

### What is known and what is new?

- Expert labelling time for clinical image data is limited but the most efficient data labelling strategy is not known.
- This study presents a novel way to label CMR images by multiple experts’ making rapid ranked comparisons.
- This is more efficient than a single expert classifying one image a time.

### What is the implication, and what should change now?

- We recommend the ‘multiple-image-ranking’ approach to label ordinal clinical images.
- This could maximise yield from clinical expert labelling time, and get more secure labels that are representative of a consensus of experts rather than a single opinion.
